# Hypercapnia-Induced Noncirrhotic Hyperammonemia (HIH): Unveiling an Unrecognized Association

**DOI:** 10.7759/cureus.90947

**Published:** 2025-08-25

**Authors:** Fortune O Alabi, Christopher O Alabi, Hadaya A Alkhateeb, Suma J Kurian, Claudia Romero, Noah B Andersen

**Affiliations:** 1 Pulmonary, Critical Care and Sleep Medicine, Florida Lung, Asthma and Sleep Specialists, Orlando, USA; 2 Internal Medicine, Florida Lung, Asthma and Sleep Specialists, Orlando, USA; 3 Pulmonary Medicine, Florida Lung, Asthma and Sleep Specialists, Orlando, USA; 4 Biology, Florida Lung, Asthma and Sleep Specialists, Orlando, USA

**Keywords:** chronic hypoxic respiratory failure, cirrhosis, hyperammonemia, hypercapnia, noncirrhotic hyperammonemia, noninvasive ventilation, portosystemic shunt

## Abstract

Hypercapnia-induced noncirrhotic hyperammonemia (HIH) presents a perplexing clinical scenario, challenging the conventional understanding of ammonia metabolism. Despite the well-established link between hyperammonemia and liver dysfunction, our recent observations revealed a novel association between hypercapnia and elevated ammonia levels in patients without chronic liver disease. This abstract encapsulates a brief report outlining five cases of HIH, shedding light on the pathophysiological mechanisms underlying this phenomenon and emphasizing the importance of heightened clinical awareness. This brief report elucidates the intricate interplay between respiratory and metabolic processes in the modulation of ammonia homeostasis. Ammonia, a neurotoxic metabolite primarily detoxified by hepatic mechanisms, has traditionally been associated with liver dysfunction. However, the emergence of HIH suggests alternative pathways influencing ammonia levels. The manuscript hypothesized possible mechanisms, and in addition, it explores the role of pulmonary vascular dynamics and congenital shunts in precipitating hyperammonemia in hypercapnic states. Recognition of this association not only prevents misdiagnosis of liver pathology but also informs targeted therapeutic interventions. Furthermore, the manuscript advocates for a paradigm shift in clinical practice, advocating for repeat ammonia measurements following the resolution of hypercapnia to ascertain the transient nature of hyperammonemia. While this report offers valuable insights into the enigmatic realm of HIH, it underscores the need for further research to elucidate the epidemiology, risk factors, and mechanistic underpinnings of this phenomenon. Future investigations could refine the mechanism and clinical significance of this association and ultimately optimize patient care in the context of hyperammonemia associated with hypercapnia.

## Introduction

Ammonia is a by-product of the metabolism of amino acids as well as other nitrogen-containing compounds [1]. It is derived from the intestinal lumen via the cleavage of urea to ammonia and carbonate by urease-producing bacteria [2]. Ammonia is neurotoxic, and at high concentrations, it can cause stupor, seizure, and coma. The level of ammonia in the body is tightly regulated by detoxication systems in the liver, primarily the urea cycle and the glutamine system. In addition, ammonia is involved in maintaining acid-base homeostasis [3]. At physiological pH, ammonia predominantly exists in its ionized form (NH4). Toxic levels of ammonia in the blood usually occur due to impaired endogenous hepatic detoxification or inherited urea cycle deficiency in one of the six key enzymes of the urea cycle or one of the three transporters that are responsible for providing ornithine, aspartate, and arginine. Valproic acid can act as a suppressor of the urea cycle and can be associated with a transient increase in ammonia levels [4]. Overgrowth of urease-producing bacteria can also lead to hyperammonemia in patients with complicated urinary tract infections (UTIs).

The most common causes of altered mental status include sepsis, metabolic changes, sedative use, hypercapnia, and hyperammonemia. Therefore, the evaluation of patients with changes in mental status usually includes, but is not limited to, checking arterial blood gas (ABG) for hypercapnia and ammonia levels. Ammonia levels are mostly checked in patients with a history of chronic liver disease or suspected hepatic insufficiency. The causes of hyperammonemia can be divided into cirrhotic and noncirrhotic. Drug use [4], urea cycle deficiency, portosystemic shunt placement [5], and complicated urinary tract infections [6] are the most common etiologies of noncirrhotic hyperammonemia. There are also well-known conditions that exacerbate hepatic encephalopathy by increasing ammonia levels and increasing nitrogen content in the gut, such as gastrointestinal bleeding. Alkalosis can also increase the amount of the gaseous form of ammonia (NH3), which is more diffusible, thereby enhancing its ability to cross the blood-brain barrier. Metabolic acidosis and hypokalemia are also associated with increased kidney production of ammonia [7,8]. Hypercapnia and hyponatremia can worsen hepatic encephalopathy through their suppressive effect on the central nervous system, but have hitherto not been associated with increased ammonia levels.

Hypercapnia is not a well-recognized cause of hyperammonemia. In this brief report, we draw the attention of the medical community to our observation of hyperammonemia in some patients with hypercapnia without underlying chronic liver disease.

Our objectives are as follows: to describe five patients with hyperammonemia and hypercapnia without underlying liver disease who presented with hypercapnia-induced noncirrhotic hyperammonemia (HIH), to propose a mechanism underlying this observation, and finally, to increase awareness of this phenomenon and to stimulate subsequent research that will further elucidate the underlying mechanism involved.

The patients included in this case series had no history of liver disease or significant renal impairment. All patients had normal liver function tests (LFTs) and kidney function at chronic kidney disease (CKD) stage 1 or 2. No evidence of cirrhosis was noted on computed tomography (CT) scans of their abdomens or liver ultrasounds. None of the patients was on medications known to cause hyperammonemia. All the patients were admitted with hypercapnic respiratory failure, and some of them experienced a change in mental status.

## Case presentation

Case 1

Our first observation of the association between hypercapnia and hyperammonemia was serendipitous. The first patient in the case series with unexplained hypercapnia at presentation was our index patient. He was sent to our clinic for evaluation of unexplained hypercapnia. He was a lifelong nonsmoker without an underlying history of any chronic lung disease. He was admitted to the hospital with an altered mental status. His toxicology test was negative, but he was severely hypercapnic, with a partial pressure of carbon dioxide (PaCO2) of 97 mmHg and an ammonia level of 103 μmol/L. He underwent extensive evaluation for liver disease at the hospital, and all the results were within the normal limits. Chest X-ray and computed tomography angiography (CTA) of the chest performed at the hospital were also normal. He was placed on noninvasive ventilation (NIV), prescribed lactulose four times daily, and discharged from the hospital for further evaluation of his hypercapnia. The hyperammonemia resolved after he was placed on NIV, and his serum ammonia levels remained normal on outpatient follow-up after lactulose was discontinued. His outpatient pulmonary function test (PFT) showed evidence of restriction with a normal diffusion capacity. The sniff test was not conclusive but was suggestive of possible bilateral diaphragmatic paralysis. Serology for myasthenia gravis was negative. Autoimmune evaluations, which included creatine phosphokinase (CPK), aldolase, and myositis-associated antibody tests, and tests for specific antibodies were all negative. The patient was suspected of having amyotrophic lateral sclerosis (ALS), which was later confirmed at the Mayo Clinic in Rochester. Subsequently, we noted additional patients who were admitted with hypercapnia and who also had elevated ammonia levels that resolved after the hypercapnia was corrected with NIV. None of the other patients in this short case series required treatment with lactulose or any other treatment for hyperammonemia.

Case 2

A 41-year-old woman with morbid obesity (BMI: 84 kg/m²), asthma, pulmonary hypertension (mean pulmonary artery pressure (PAP): 52 mmHg, pulmonary artery wedge pressure (PAWP): 13, pulmonary vascular resistance (PVR): 7), obesity hypoventilation syndrome (OHS), and untreated obstructive sleep apnea (OSA) presented with shortness of breath and mild confusion. She was noncompliant with bilevel positive airway pressure (BIPAP) due to intolerance. Initial laboratory work revealed an elevated bicarbonate of 35 and serum ammonia of 56 µmol/L. Liver function tests (LFTs), thyroid-stimulating hormone (TSH) test, and toxicology were unremarkable. After the initiation of BIPAP overnight, her ammonia levels normalized by the next morning. No hepatic pathology was identified.

Case 3

A 76-year-old man with coronary artery disease, chronic obstructive pulmonary disease (COPD), and obesity (BMI: 43 kg/m²) was admitted with acute on chronic dyspnea. He was diagnosed with a COPD exacerbation and acute hypercapnic respiratory failure. ABG revealed a pH of 7.22 and PCO₂ of 64 mmHg. Serum ammonia was elevated at 78 µmol/L. After the initiation of noninvasive ventilation (NIV), his PCO₂ and ammonia levels improved significantly, with the ammonia decreasing to 21 µmol/L. LFTs, urinalysis, urine toxicology, and medication review were unremarkable. Ammonia remained within normal limits throughout hospitalization.

Case 4

A 53-year-old man with morbid obesity, asthma, and OSA presented with shortness of breath and was diagnosed with asthma exacerbation and acute on chronic hypercapnic respiratory failure. ABG confirmed chronic hypercapnia, and serum ammonia was elevated at 68 µmol/L. LFTs and TSH were normal, and the patient's urinalysis was unremarkable. After treatment with BIPAP, which was well-tolerated, his ammonia levels normalized. He was discharged with BIPAP and had no recurrence of hyperammonemia at follow-up.

Case 5

A 90-year-old woman with obesity (BMI: 32.4 kg/m²), congestive heart failure, CKD stage 2, and severe pulmonary fibrosis presented with altered mental status and shortness of breath. ABG showed profound hypercapnia (PCO₂: 130 mmHg) and a pH of 7.18. CTA was negative for pulmonary embolism (PE) but confirmed severe fibrosis. Serum ammonia was 67 µmol/L and improved to 19 µmol/L after >12 hours on NIV. Echocardiography showed right ventricular systolic pressure (RVSP) of 60 mmHg. She had lifelong biomass exposure but no history of asthma or COPD. Due to advanced age and prognosis, hospice care was pursued; she passed away two days after discharge.

Table 1 summarizes the five cases discussed above.

**Table 1:** Case series table Units and normal values BMI: kg/m² Ammonia (10-47 µmol/L), determined at our hospital laboratory pH: 7.35-7.45 PaCO2: 35-45 mmHg RHC: right heart catheterization, BMI: body mass index, CAD: coronary artery disease, COPD: chronic obstructive pulmonary disease, RA: rheumatoid arthritis, ALS: amyotrophic lateral sclerosis, MVR: mitral valve replacement, OSA: obstructive sleep apnea, OHS: obesity hypoventilation syndrome, HTN: hypertension, NIV: noninvasive ventilation, NH3: ammonia, PaCO2: partial pressure of carbon dioxide

Age	Sex	Race	Clinical history	BMI (kg/m²)	pH (7.35-7.45)	PCO₂ (35-45 mm Hg)	Ammonia (10-47 µmol/L)	Post-NIV ammonia (10-47 µmol/L)
73	Male	White	ALS, HTN, and CAD	27.8	7.33	97	103	45
41	Female	Black	Asthma, pulmonary hypertension, morbid obesity with a BMI of 84 kg/m², RHC: mean pulmonary artery pressure of 52, pulmonary artery wedge pressure of 13, pulmonary vascular resistance of 7, and RAP of 22	84	7.37	60	56	19
76	Male	White	History of CAD, COPD, and obesity	43.4	7.22	64	78	21
53	Male	Hispanic	Asthma and OSA	60.3	7.31	77	68	20
90	Female	Hispanic	CHF and RA	32.4	7.18	130	67	19

## Discussion

The first description of hypercapnic hyperammonemia in the literature can be found in an abstract by Stepanek at the Lung Conference in 1977 [9]. Subsequently, a study was conducted on mongrel dogs in 1979 to understand the underlying mechanism involved. In this study, portal vein vasoconstriction was induced using 1 mg/kg oxprenolol in anesthetized and artificially ventilated dogs. Artificial hypercapnia results in an increase in arterial ammonia levels by opening the portal to the systemic venous shunt. The induction of portal vasoconstriction by oxprenolol eliminated hyperammonemia during normoxia and hypercapnia. On the other hand, the presence of portal hypertension via the vasodilating effect of phentolamine resulted in increased arterial ammonia in the presence or absence of hypercapnia. This study suggested that portosystemic shunts secondary to hypercapnia may be a contributing factor to arterial hyperammonemia independent of the presence of portal hypertension. Elevated pulmonary pressure may indirectly increase posthepatic portal hypertension and thereby lead to portosystemic shunting.

Huang and El Younis reported a case of noncirrhotic hyperammonemia secondary to hypercapnic respiratory failure in a 69-year-old woman with severe COPD with no evidence of liver cirrhosis, which was attributed to a functional portosystemic shunt from high right-sided pressure [10]. It is possible that elevated right-sided heart pressure can contribute to hyperammonemia in some of these patients. There is also a possibility of congenital portosystemic shunts in some of these patients. A case of patent ductus venosus resulting in nonhepatic hyperammonemia was reported by Nakayama et al. in a 47-year-old man with asthma and respiratory failure who developed a change in mental status from severe hyperammonemia [11]. Finally, we cannot exclude the possibility that urea cycle disorders are a contributing factor. Carbamoyl phosphate synthetase 1 (CPS-1) dysfunction was reported in a 65-year-old woman postmortem [12]. In our experience, the resolution of hyperammonemia usually occurred within 12-24 hours in all patients who were treated with NIV.

## Conclusions

Awareness of the association between hypercapnia and hyperammonemia will mitigate unnecessary treatment of patients with liver disease/liver cirrhosis and unnecessary diagnostic testing for hyperammonemia without at least first addressing hypercapnia and repeat blood ammonia levels when hypercapnia has resolved. Our findings suggest that a repeat blood ammonia level measurement after rectifying hypercapnia is valuable in cases in which the ammonia level is elevated in the setting of hypercapnia.

This observation is only hypothetical, and further studies are needed to identify the incidence and risk factors and, most importantly, to elucidate the underlying mechanism involved.
